# Human and Mouse Nephrin and Their Interactions With 13 Proteins: An In Silico Study

**DOI:** 10.7759/cureus.66332

**Published:** 2024-08-06

**Authors:** Radhakrishnan Narayanaswamy, Vemugadda Harika, Vasantha-Srinivasan Prabhakaran

**Affiliations:** 1 Department of Biochemistry, Saveetha Medical College and Hospital, Saveetha Institute of Medical and Technical Sciences (Deemed to be University), Chennai, IND; 2 Department of Bioinformatics, Saveetha School of Engineering, Saveetha Institute of Medical and Technical Sciences (Deemed to be University), Chennai, IND

**Keywords:** mouse nephrin, good health and well- being, cystatin c, dynamin, podocin, podocyte, human nephrin

## Abstract

Background

Human nephrin (hNeph) (podocyte protein) has been known to be involved in both the formation and maintenance of the slit diaphragm (SD) and also acts as a hub protein in the podocyte by modulating cell polarity, cell survival, cell adhesion, cytoskeletal organization, mechano-sensing, and SD turn-over.

Methodology

In the present investigation, we aimed to analyse the hNeph and mouse nephrin (mNeph) and their interactions with 13 proteins using the molecular docking method. The 13 selected human proteins which include matrix metalloproteinases (MMP 2 and 9), retinol-binding proteins (RBP 3 and 4), kallikrein 1 (KLK 1), uromodulin, insulin-like growth factor binding protein 7 (IGFBP7), cystatin C, podocin, beta arrestin 1, vang-like protein 2 (VANGL2), dynamin 1, and tensin-like C1 domain-containing phosphatase (TENC1) were studied on the docking analysis of hNeph and mNeph by using the HDOCK (protein-protein) docking method. In addition, the physicochemical (PC) properties of 15 proteins were performed using the ProtParam web server.

Results

In the present investigation, five chosen human proteins, namely, IGFBP7, cystatin C, podocin, VANGL2, and TENC1, have exhibited theoretical isoelectric point (PI) values greater than 7.0. The protein-protein docking analysis has shown that hKLK and hVANGL2 exhibited the maximum docking score of -206.39 kcal/mol and -329.28 (kcal/mol) with the target proteins mNeph and hNeph, respectively.

Conclusions

Thus, the current finding highlights the interactions of hNeph and mNeph with 13 chosen proteins, which may help in renal disease management.

## Introduction

Nephrin is an essential component of the extracellular portion of the slit diaphragm (SD), which is situated between the basement form calyx of the glomerular filtration barrier and the foot process of podocytes along with endothelial cells. It is a transmembrane protein, with a molecular weight of approximately 180 kDa, and has 1241 amino acids [1-3]. It plays a key role in the SD of podocytes, and it forms a molecular sieve that permits the passage of tiny molecules while blocking the loss of vital proteins and blood cells into the urine, hence enhancing the glomerular filtration barrier's selective permeability. However, in the case of hypertension, angiotensin II acts on the podocyte AT1-receptor to induce nephrin-β-arrestin2 binding and nephrin endocytosis, resulting in the enhancement of the glomerular permeability and in turn contributing to albuminuria in mice. The interaction between nephrin on podocytes and various other proteins, including those on endothelial cells and within the basement membrane, is critical for the proper functioning of the glomerular basement membrane (GBM). Disruption or dysfunction of nephrin can lead to various kidney diseases [4,5]. With regard to diabetic nephropathy (DN), alterations in nephrin expression and its urinary excretion have been observed in the early stages. Numerous studies have reported significant increased urinary nephrin concentration with DN, particularly in microalbuminuria or overt proteinuria [6-8]. Moreover, urinary nephrin levels have been correlated with the severity of renal histopathological changes and the progression of DN. Furthermore, urinary nephrin identification also plays a significant role in the early detection of severe pre-eclampsia [9].

Similarly, elevated urinary matrix metalloproteinase 9 (MMP 9) levels in DN patients have been reported [10]. Hirata and colleagues have reported that the activation of the MMP 2 enzyme is involved in the pathogenesis of both hypertension and DN [11]. Tsai and coworkers have demonstrated that the urinary retinol-binding protein 4 (RBP 4) levels have been increased in severe non-alcoholic fatty liver disease with both hypertension and proteinuria conditions [12]. The other protein plasma kallikrein (KLK) has been reported to possess a protective effect on renal function especially in type 1 diabetes mellitus (DM) [13]. Uromodulin has been reported as the principal urinary protein in healthy persons [14]. Wang and colleagues have reported that the insulin-like growth factor binding protein 7 (IGFBP7) has been utilized as one of the biomarkers for the early diagnosis and prognosis of acute renal injury [15]. Benoit and coworkers have reported the serum cystatin C as a biomarker for the early diagnosis of chronic renal disease [16]. ElShaarawy and colleagues have demonstrated the urinary podocin as a novel biomarker for the early diagnosis of diabetic renal disease in type 2 DM [17]. Xu and colleagues have reported that the beta arrestin 1 deficiency results in reduced renal fibrosis under the experimental mice model [18]. Rocque and coworkers have demonstrated the physiological role of vang-like protein 2 (VANGL2) in the maintenance of glomerular structure as well as perm selectivity in the adult mice kidney [19]. Khalil and colleagues have reported that the dynamin expression level has been elevated in human proteinuric kidney diseases [20]. Lee and colleagues have reported that the tensin-like C1 domain-containing phosphatase (TENC1) has been known to be involved in the pathogenesis of diabetic kidney disease by inducing podocyte hypertrophy under hyperglycemic condition [21].

The interaction between nephrin on podocytes and various other proteins is important for renal physiological function, hence the present investigation where we selected 13 human proteins which include MMP 2 and 9, RBP 3 and 4, KLK 1, uromodulin, IGFBP7, cystatin C, podocin, beta arrestin 1, VANGL2, dynamin 1, and TENC1. These 13 human proteins were studied on the docking analysis of human nephrin (hNeph) and mouse nephrin (mNeph) by using the HDOCK (protein-protein) docking method, in order to know their interactions and binding affinities.

## Materials and methods

Preparation of target ligands (proteins)

The three-dimensional (3D) structures of (a) human MMP 2 (hMMP 2) (1QIB.pdb), (b) human MMP 9 (hMMP 9) (4H1Q.pdb), (c) human RBP 3 (hRBP 3) (1GGL.pdb), (d) human RBP 4 (hRBP 4) (6QBA.pdb), (e) human KLK 1 (hKLK 1) (1SPJ.pdb), (f) human uromodulin (hUromodulin) (4WRN.pdb), (g) human IGFBP 7 (hIGFBP 7) (8IVD.pdb), (h) human cystatin C (hCystatin C) (3QRD.pdb), (i) human podocin (hPodocin) (AlphaFold DB: Q9NP85), (j) human beta arrestin 1 (hBeta arrestin 1) (8AS4.pdb), (k) human VANGL2 (hVANGL2) (AlphaFold DB: Q9ULK5), (l) human dynamin 1 (hDynamin 1) (2X2E.pdb), and (m) human TENC1 (hTENC1) (3HQC.pdb) were downloaded from the Protein Data Bank (a-h, j, l, and m) and AlphaFold database (i and k). A chain of all 13 human proteins was prepared independently by carefully deleting other chains and heteroatoms including crystallographically observed water by utilizing the UCSF Chimera free software [22].

Preparation of target or receptor proteins (hNeph and mNeph)

The 3D structure of hNeph (AlphaFold DB: 060500) and mNeph (5ZYS.pdb) was downloaded from the Protein Data Bank (hNeph) and AlphaFold database (mNeph), respectively. A chain of hNeph and B chain of mNeph were prepared independently by carefully deleting other chains and heteroatoms including crystallographically observed water by applying the UCSF Chimera free software [22].

Physicochemical (PC) property analysis

ProtParam web server was utilized to determine the PC properties of 13 target ligands (proteins) and two target receptors (proteins), namely, hMMP 2, hMMP 9, hRBP 3, hRBP 4, hKLK, hUromodulin, hIGFBP 7, hCystatin C, hPodocin, hBeta arrestin 1, hVANGL2, hDynamin 1, hTENC1, hNeph, and mNeph. A chain amino acid (single letter) sequence of 15 proteins (except mNeph, which is a B chain) was provided separately as an input file for PC analysis [23,24].

Docking study

The docking analysis was performed for 13 selected target ligands with that of hNeph and mNeph (target receptor proteins) individually using the protein-protein (HDOCK) method [24].

## Results

In the present investigation, Table 1 represents the PC properties of 15 proteins, where five (33.33%) chosen human proteins, namely, hIGFBP7, hCystatin C, hPodocin, hVANGL2, and hTENC1, have exhibited a theoretical isoelectric point (PI) value greater than 7.0. On the other hand, 10 (66.66%) chosen proteins (hMMP 2, hMMP 9, hRBP 3, hRBP 4, hKLK, hUromodulin, hBeta arrestin 1, hDynamin 1, hNeph, and mNeph) have shown a theoretical PI value lesser than 7.0. Interestingly, seven chosen proteins (hMMP 2, hMMP 9, hRBP 3, hRBP 4, hKLK, hUromodulin, and hBeta arrestin 1) have shown an instability index value lesser than 40 (as shown in Table 1).

**Table 1:** The PC properties of 15 proteins using the ProtParam free web server. mNeph: mouse nephrin; hNeph: human nephrin; hMMP 2: human matrix metalloproteinase 2; hMMP 9: human matrix metalloproteinase 9; hRBP 3: human retinol-binding protein 3; hRBP 4: human retinol-binding protein 4; hKLK 1: human kallikrein 1; hUromodulin: human uromodulin; hIGFBP 7: human insulin-like growth factor binding protein 7; hCystatin C: human cystatin C; hPodocin: human podocin; hBeta arrestin 1: human beta arrestin 1; hVANGL2: human vang-like protein 2; hDynamin 1: human dynamin 1; hTENC1: human tensin-like C1 domain-containing phosphatase; MW^■^: molecular weight of protein; P1^◊^: theoretical isoelectric point of protein; NR^◘^: total number of negatively charged residues present in the protein; PRׁ: total number of positively charged residues present in the protein; Ext.co1^▼^: extinction coefficient assuming all pairs of Cys residues form cystines; Ext.co2^▲^: extinction coefficient assuming all Cys residues are reduced; GRAVY^*^: grand average of hydropathicity of the protein; PC: physicochemical

Proteins	MW^■^	P1^◊^	NR^◘^	PR^ׁ^	Ext.co1^▼^	Ext.co2^▲^	Instability index	Aliphatic index	GRAVY^*^
mNeph	1180.4	6.75	1	1	-	-	58.29	146.00	0.520
hNeph	134742.2	5.42	139	106	166490	165240	46.04	86.74	-0.246
hMMP 2	17936.9	4.85	25	14	32430	-	15.46	69.75	-0.461
hMMP 9	17911.9	5.12	22	10	33920	-	27.70	77.50	-0.281
hRBP 3	15781.0	5.97	20	17	24075	23950	30.16	86.42	-0.484
hRBP 4	21225.8	5.27	28	24	34295	33920	33.50	65.35	-0.592
hKLK 1	26405.6	4.62	34	13	46575	45950	39.66	79.79	-0.229
hUromodulin	76456.7	5.60	82	67	96230	95230	24.75	78.94	-0.258
hIGFBP7	50266.4	7.62	47	49	46390	43890	41.44	53.58	-0.443
hCystatin C	13333.1	8.75	12	15	11710	11460	45.63	64.17	-0.507
hPodocin	42200.9	8.95	45	51	24325	23950	53.38	93.47	-0.170
hBeta arrestin-1	47051.6	5.84	66	59	19495	19370	36.26	86.67	-0.515
hVANGL2	59714.5	9.27	61	72	67060	66810	48.41	90.36	-0.297
hDynamin 1	39333.0	5.89	48	43	10555	10430	40.62	97.45	-0.310
hTENC1	17191.5	9.18	12	16	14105	13980	46.52	72.10	-0.315

The HDOCK (protein-protein) docking analysis showed that the hKLK 1 has exhibited the highest docking score of -206.39 kcal/mol with the target protein (mNeph). On the other hand, hPodocin has exhibited the least docking score (-164.41 kcal/mol) with the target protein (mNeph) as shown in Table 2. The HDOCK docking score results have exhibited the following order: hKLK 1 (-206.39 kcal/mol) ˂ hMMP 9 (-205.44 kcal/mol) ˂ hTENC1 (-186.64 kcal/mol) ˂ hDynamin 1 (-186.17 kcal/mol) ˂ hRBP 4 (-180.22 kcal/mol) ˂ hVANGL2 (-177.26 kcal/mol) ˂ hMMP 2 (-176.95 kcal/mol) ˂ hUromodulin (-169.37 kcal/mol) ˂ hIGFBP 7 (-167.09 kcal/mol) ˂ hRBP 3 (-166.45 kcal/mol) ˂ hBeta arrestin 1 (-166.14 kcal/mol) ˂ hCystatin C (-164.90 kcal/mol) ˂ hPodocin (-164.41 kcal/mol).

**Table 2:** The protein-protein docking (mNeph docked with 13 interested proteins) using the HDOCK free online server. hMMP 2: human matrix metalloproteinase 2; hMMP 9: human matrix metalloproteinase 9; hRBP 3: human retinol-binding protein 3; hRBP 4: human retinol-binding protein 4; hKLK 1: human kallikrein 1; hUromodulin: human uromodulin; hIGFBP 7: human insulin-like growth factor binding protein 7; hCystatin C: human cystatin C; hPodocin: human podocin, hBeta arrestin 1: human beta arrestin 1; hVANGL2: human vang-like protein 2; hDynamin 1: human dynamin 1; hTENC1: human tensin-like C1 domain-containing phosphatase; ^■^bond distance (Aᵒ): less than 4.0 Aᵒ; mNeph: mouse nephrin

S. no.	Protein name	HDOCK docking score (-kcal/mol)	Ligand-receptor interface residue pair(s)	Bond distance^■^ (A^ᵒ^)
1	hMMP 2	176.95	Tyr155-Phe1249	2.60
			Tyr155-Arg1252	2.16
			Pro156-Phe1249	3.97
			Asp161-Val1256	3.98
			Gly162-Leu1255	3.73
			Gly162-Val1256	2.84
			Leu163-Val1256	3.69
			Leu164-His1254	3.06
			Leu164-Leu1255	3.91
			Leu164-Val1256	0.83
			Ala165-Gly1253	2.58
			Ala165-His1254	3.06
			His166-Gly1253	3.65
			Tyr193-Val1256	2.65
			His201-Gly1253	3.57
			His201-His1254	3.52
			Glu202-Gly1253	3.56
			Glu202-His1254	3.03
			His205-Leu1247	2.96
			Glu210-Leu1247	2.32
			His211-Leu1247	3.41
			His211-Leu1251	2.11
			His211-Gly1253	3.59
			Pro221-His1254	3.95
			Pro221-Leu1255	2.86
			Ile222-His1254	3.75
			Ile222-Leu1255	2.44
			Tyr223-His1254	2.22
			Tyr223-Leu1255	2.62
			Tyr223-Val1256	3.75
2	hMMP 9	205.44	Tyr149-Phe1249	2.29
			Tyr149-Arg1252	2.69
			Gly186-Val1256	2.88
			Leu188-His1254	3.56
			Leu188-Val1256	2.12
			Ala189-Arg1252	3.91
			Ala189-Gly1253	2.49
			Ala189-His1254	3.63
			His190-Arg1252	3.55
			His190-Gly1253	3.57
			Ala191-Arg1252	3.35
			Phe192-Arg1252	3.74
			Pro193-Leu1247	3.52
			Tyr218-Val1256	3.26
			His226-Gly1253	3.25
			His226-His1254	3.48
			Gln227-Gly1253	3.04
			Gln227-His1254	2.91
			His230-Leu1247	2.44
			His230-Leu1251	3.81
			His230-Gly1253	3.79
			Asp235-Leu1247	3.67
			His236-Leu1251	2.33
			His236-Gly1253	3.57
			Pro246-Leu1255	1.97
			Met247-His1254	3.14
			Met247-Leu1255	2.64
			Tyr248-His1254	2.14
			Tyr248-Leu1255	2.13
			Tyr248-Val1256	2.81
3	hRBP 3	166.45	Tyr19-His1254	3.46
			Leu20-His1254	3.02
			Leu23-His1254	3.59
			Ile25-Gly1253	2.66
			Ile25-His1254	2.87
			Ser26-Arg1252	3.92
			Val29-Arg1252	3.65
			Val29-Gly1253	2.83
			Val29-His1254	3.60
			Leu36-Val1256	2.10
			Pro38-Val1256	3.71
			Thr53-Val1256	2.69
			Leu54-Val1256	3.64
			Ser55-Val1256	2.84
			Arg58-Phe1249	3.25
			Arg58-Glu1250	2.63
			Arg58-Leu1251	3.75
			Arg58-Arg1252	2.64
			Arg58-Leu1255	2.39
			Arg58-Val1256	2.02
			Asn59-Leu1255	2.36
			Asn59-Val1256	3.49
			Tyr60-Leu1255	2.97
			Arg75-Leu1251	2.68
			Ser76-Leu1255	2.94
			Val77-Leu1251	3.97
			Val77-Gly1253	1.65
			Val77-His1254	2.75
			Val77-Leu1255	3.51
			Asp78-Leu1247	3.51
			Asp78-Gly1253	3.85
4	hKLK 1	206.39	Phe40-His1254	2.20
			Gln41-Arg1252	3.99
			Gln41-Gly1253	2.16
			Gln41-His1254	3.25
			His57-Leu1247	3.35
			His57-Arg1252	3.10
			Leu95-Phe1249	2.84 and 3.33
			Leu95-Glu1250	3.95
			Leu95-Arg1252	2.78 and 2.95
			Tyr99-Leu1247	3.18
			Tyr99-Pro1248	3.51
			Tyr99-Arg1252	3.07
			Phe149-Leu1251	3.75
			Phe151-His1254	2.78
			Phe151-Leu1255	3.26
			Val192-Leu1251	2.53
			Val192-Gly1253	3.51
			Ser195-Leu1247	2.53
			Ser214-Leu1247	2.94
			Trp215-Leu1247	3.50
			Val218-Leu1251	3.04
5	hUromodulin	169.37	Asp38-Leu1255	2.75
			Asp38-Val1256	3.66
			Lys39-Val1256	3.56
			His63-Phe1249	3.61
			Pro64-Phe1249	3.89
			Asp65-Phe1249	3.51
			Asp65-Arg1252	1.75
			Lys66-Arg1252	3.69
			Lys66-Gly1253	3.93
			Glu68-Gly1253	3.82
			Glu68-His1254	3.27
			Trp86-His1254	3.03
			Trp86-Val1256	3.64
			Ala87-Val1256	3.70
			Glu135-Val1256	2.08
			Glu177-Gly1253	2.32
			Glu177-His1254	2.65
			Glu177-Leu1255	3.78
			Tyr179-His1254	3.73
			Tyr179-Leu1255	3.60
			Tyr179-Val1256	3.91
			Phe180-Leu1255	2.55
			Asp233-Leu1251	2.71
			Tyr234-Leu1251	3.70
			Tyr234-Leu1255	2.98
			Ser235-Glu1250	3.68
			Ser235-Leu1251	3.71
			Trp254-Leu1255	3.91
			Trp254-Val1256	3.03
			Trp364-His1254	3.61
			Ile402-Leu1247	3.21
6	hRBP 4	180.22	Phe36-His1254	3.07
			Phe36-Leu1255	3.97
			Leu37-His1254	2.92
			Ile41-His1254	3.99
			Phe45-Val1256	3.41
			Ala57-Val1256	1.88
			Met73-His1254	2.75
			Phe77-Val1256	3.48
			Met88-Leu1255	1.47
			Met88-Val1256	1.65
			Tyr90-Leu1251	3.76
			Tyr90-Arg1252	2.15
			Tyr90-Gly1253	3.04
			Tyr90-His1254	3.33
			Tyr90-Leu1255	3.66
			Gln98-Gly1253	3.66
			Lys99-Phe1249	2.73
			Lys99-Arg1252	2.55
			Gly100-Phe1249	2.84
			Gly100-Arg1252	3.65
			Asn101-Phe1249	3.88
			Asn101-Glu1250	2.35
			Asp102-Glu1250	2.77
			Asp102-Leu1251	3.91
			Asp102-Leu1255	2.83
			Asp103-Leu1255	2.95
			His104-Leu1255	3.95
			His104-Val1256	3.20
			Gln117-Leu1255	3.78
			Ser119-Leu1255	3.33
			Arg121-Leu1251	2.33
			Arg121-Gly1253	2.96
			Asp131-Leu1251	3.92
			Tyr133-Leu1255	1.19
			Phe135-His1254	2.56
			Phe135-Leu1255	3.56
7	hIGFBP 7	167.09	Leu191-Leu1247	2.73
			Ala192-Gly1253	3.54
			Leu193-Leu1247	3.21
			Leu193-Arg1252	3.04
			Leu193-Gly1253	3.84
			Gly195-Arg1252	3.43
			Pro196-Arg1252	3.83
			Gly197-Leu1247	2.92
			Gly197-Arg1252	3.00
			Gln198-Leu1247	3.18
			Gln198-Pro1248	3.54
			Val199-Leu1247	2.69
			Tyr201-Leu1247	3.15
			Ser210-Leu1247	3.34
			Ser210-Pro1248	3.52
			Ser210-Leu1251	3.90
			Ser211-Leu1251	2.87
			Phe266-Phe1249	2.93
			Phe266-Arg1252	2.34
			Asn267-Phe1249	3.22
			Asn267-Arg1252	3.55
			Gly270-Gly1253	3.97
			His272-His1254	2.51
			His272-Val1256	3.06
8	hCystatin C	164.90	Val10-Glu1250	2.35
			Val10-Leu1251	2.37
			Val10-Arg1252	3.76
			Val10-Leu1255	3.40
			Gly11-Leu1251	3.19
			Gly11-Gly1253	3.20
			Gly11-Leu1255	3.65
			Gly12-Leu1255	2.97
			Met14-Leu1251	3.86
			Gln55-His1254	2.77
			Gln55-Leu1255	3.64
			Ile56-Leu1247	2.86
			Ile56-Leu1251	3.70
			Ile56-Gly1253	2.46
			Ile56-His1254	3.01
			Val57-His1254	3.33
			Ala58-Gly1253	3.31
			Ala58-His1254	3.47
			Tyr102-His1254	3.92
			Trp106-Phe1249	3.55
			Trp106-Arg1252	2.74
			Trp106-Gly1253	2.98
			Gln107-His1254	2.74
			Gln107-Leu1255	3.58
			Gln107-Val1256	3.14
			Gln107-Val1256	3.46
9	hPodocin	164.41	Pro89-Pro1248	3.87
			Pro89-Arg1252	3.11
			Glu90-Leu1247	3.92
			Glu90-Arg1252	2.78
			Glu91-Arg1252	3.68
			Gly92-Phe1249	3.08
			Thr93-Phe1249	2.93
			Leu142-Leu1251	3.80
			Leu143-Glu1250	3.04
			Leu143-Leu1255	3.08
			Pro144-Glu1250	3.32
			Pro144-Leu1253	3.31
			Gly145-Glu1250	3.10
			Gly145-Leu1251	3.42
			Gly145-Arg1252	3.55
			Gly145-Gly1253	3.39
			Gly145-Leu1255	3.81
			Arg146-Leu1247	3.66
			Arg146-Leu1251	3.28
			Arg146-Gly1253	2.49
			Arg146-Leu1255	3.11
			Lys148-Leu1255	3.23
			Lys148-Val1256	3.13
			Ser201-Leu1251	2.38
			Leu202-Leu1251	3.71
10	hBeta arrestin	166.14	Thr56-Phe1249	2.82
			Ala60-Leu1255	3.27
			Glu66-His1254	3.21
			Arg76-Glu1250	2.66
			Arg76-Leu1255	2.44
			Asp78-Glu1250	2.75
			Val81-Glu1250	3.49
			Asn83-Phe1249	3.51
			Asp143-Val1256	3.25
			Glu145-His1254	3.97
			Glu145-Val1256	3.94
			Lys147-Phe1249	3.24
			Phe149-Phe1249	2.72
			Leu154-Arg1252	3.31
			Glu155-Arg1252	3.85
			Glu156-Arg1252	3.13
			Lys157-Leu1247	3.57
			Lys157-Arg1252	3.39
			Ile158-Phe1249	2.79
			Arg165-Arg1252	3.67
			Arg165-Gly1253	3.22
			Arg165-His1254	3.37
			Val167-Val1256	2.91
11	hVANGL2	177.26	Phe120-Leu1247	2.70
			Phe120-Arg1252	3.51
			Leu124-Leu1247	3.12
			Leu124-Arg1252	3.86
			Leu124-Gly1253	2.95
			Leu127-His1254	3.42
			Leu128-His1254	3.87
			Pro131-His1254	3.21
			Glu146-Val1256	3.81
			Phe149-His1254	3.29
			Phe149-Val1256	2.84
			Ala153-Phe1249	3.12
			Ala153-Arg1252	3.44
			Phe154-Phe1249	3.47
			Leu156-Arg1252	3.36
			Leu157-Phe1249	3.72
			Leu157-Arg1252	2.44
			Leu160-Arg1252	3.41
			Tyr212-His1254	2.72
12	hDynamin 1	186.17	Gln198-Phe1249	2.85
			Gln198-Glu1250	2.59
			Leu220-Val1256	3.82
			Asn222-Val1256	3.38
			Arg228-Glu1250	3.05
			Arg228-Leu1255	2.07
			Arg229-Phe1249	2.41
			Arg229-Glu1250	3.28
			Arg229-Leu1255	3.55
			Arg229-Val1256	3.30
			Gly230-Leu1255	3.76
			Gly230-Val1256	2.08
			Tyr231-Val1256	3.06
			Ile232-Val1256	3.45
			Arg271-His1254	2.49
			Arg271-Leu1255	3.37
			Tyr276-His1254	3.04
			Tyr276-Val1256	2.71
			Gln284-Phe1249	3.97
			Gln284-Arg1252	3.89
			Asn287-Phe1249	3.70
			Asn287-Arg1252	2.81
			Asn287-Gly1253	3.96
			His288-Phe1249	3.51
			Asp291-Phe1249	2.82
			Asp291-Arg1252	2.44
13	hTENC1	186.64	Tyr1280-Gly1253	3.35
			Tyr1280-His1254	3.66
			Ser1283-His1254	3.41
			Thr1310-Leu1247	2.28
			Ala1312-Arg1252	2.90
			Asp1327-Arg1252	2.86
			Leu1332-Phe1249	3.40
			Phe1333-Phe1249	3.05
			Phe1334-Phe1249	3.71
			Phe1334-Arg1252	3.53
			Phe1334-Gly1253	2.87
			Phe1334-His1254	3.53
			Arg1336-His1254	2.50
			Arg1336-Leu1255	3.26
			Arg1336-Val1256	3.15
			Tyr1338-His1254	3.23
			Lys1372-His1254	3.29
			Lys1372-Leu1255	2.38
			Lys1372-Val1256	2.98
			Pro1373-Val1256	3.22
			Gly1374-Val1256	3.33
			Val1380-His1254	3.00
			His1382-His1254	2.81

Similarly, the HDOCK (protein-protein) docking analysis showed that the hVANGL2 has exhibited the highest docking score of -329.28 kcal/mol with the target protein (hNeph). On the other hand, hRBP 3 has exhibited the least docking score of -229.32 kcal/mol with the target protein (hNeph) as shown in Table 3. The HDOCK docking score results have exhibited the following order: hVANGL2 (-329.28 kcal/mol) ˂ hIGFBP 7 (-323.81 kcal/mol) ˂ hPodocin (-305.82 kcal/mol) ˂ hCystatin C (-295.47 kcal/mol) ˂ hMMP 2 (-281.15 kcal/mol) ˂ hTENC1 (-269.73 kcal/mol) ˂ hMMP 9 (-269.31 kcal/mol) ˂ hUromodulin (-268.65 kcal/mol) ˂ hDynamin 1 (-262.85 kcal/mol) ˂ hBeta arrestin 1 (-254.64 kcal/mol) ˂ hKLK 1 (-250.66 kcal/mol) ˂ hRBP 4 (-234.62 kcal/mol) ˂ hRBP 3 (-229.32 kcal/mol).

**Table 3:** The protein-protein docking (hNeph docked with 13 interested proteins) using the HDOCK free online server. hMMP 2: human matrix metalloproteinase 2; hMMP 9: human matrix metalloproteinase 9; hRBP 3: human retinol-binding protein 3; hRBP 4: human retinol-binding protein 4; hKLK 1: human kallikrein 1; hUromodulin: human uromodulin; hIGFBP 7: human insulin-like growth factor binding protein 7; hCystatin C: human cystatin C; hPodocin: human podocin; hBeta arrestin 1: human beta arrestin 1; hVANGL2: human vang-like protein 2; hDynamin 1: human dynamin 1; hTENC1: human tensin-like C1 domain-containing phosphatase; ^■^bond distance (Aᵒ): less than 3.0 Aᵒ; hNeph: human nephrin

S. no.	Protein name	HDOCK docking score (-kcal/mol)	Ligand-receptor interface residue pair(s)	Bond distance^■^ (A^ᵒ^)
1	hMMP 2	281.15	Arg100-Pro142	2.97
			Ile102-Glu143	1.81
			Tyr104-Met147	2.84
			Pro110-Met147	2.11
			Asp114-Thr149	2.78
			Arg119-Pro1147	2.97
			His136-Pro142	2.96
			His136-Thr149	2.67
			Asp137-Thr141	2.65
			Asp137-Asn159	2.42
			Lys160-Leu829	2.91
			Asp161-Asp747	2.63
			Leu188-Phe1145	2.40
			Gly189-Leu1142	2.96
			Lys190-Arg827	2.88
			Lys190-Arg827	2.59
			Val191-Gln815	2.68
			Val191-Leu829	2.82
			Tyr228-Phe1145	2.18
			Phe232-Gln1148	2.92
2	hMMP 9	269.31	Gln126-Gln213	2.27
			Gln126-Leu214	1.79
			Asn127-Arg212	2.54
			Ala150-Thr999	2.27
			Tyr160-Ser231	0.88
			Arg162-Gly177	2.74
			Asp206-Arg212	2.87
			Asp207-Thr294	2.29
			Lys258-Gln1040	2.81
			Arg265-His955	2.72
			Gly269-Thr1001	2.19
3	hRBP 3	229.32	Pro2-Glu583	2.98
			Asn3-Arg620	2.97
			Thr5-Arg620	2.48
			Arg9-Ser671	2.54
			Val11-Gly650	2.81
			Val11-Glu651	2.19
			Ser12-Glu651	2.94
			Val29-Ser1128	2.97
			Lys37-Ser669	2.37
			Phe57-Ser1127	2.91
			Arg58-Ser1127	2.49
			Arg131-Tyr644	2.91
			Val133-His617	2.06
			His134-Arg639	0.74
4	hKLK 1	250.66	His35-Trp707	2.77
			Phe36-Glu663	1.68
			Phe36-Arg697	2.86
			Phe36-His705	2.72
			Ser38-Arg697	1.95
			Thr39-Arg697	2.85
			Gln41-Trp707	2.63
			His48-Thr287	2.74
			Arg49-Pro284	2.32
			Met95A-Gln661	2.78
			Leu95D-Asn708	2.82
			Thr241-Gln1148	2.27
			Glu244-Gln1148	2.41
			Ser246-Thr287	2.51
5	hUromodulin	268.65	Asp463-Arg256	2.46
			Arg465-Arg256	2.65
			Arg465-Lys426	2.62
			Arg465-Glu427	2.37
			Val492-Ser260	2.67
			Leu493-Pro248	1.54
			Thr494-Glu262	3.00
			Thr494-Pro264	2.78
			Arg495-Glu246	2.04
			Asn496-Glu246	2.67
			Glu497-Arg299	2.71
			Pro566-Val244	2.71
			Pro566-Arg268	2.65
			Phe584-Arg268	2.91
			Tyr586-Arg268	3.00
			Asp632-Ser208	2.93
			Asp632-Asn211	2.25
			Ser633-Phe238	2.65
			Ser633-Gly269	2.85
			Ser633-Gly270	2.86
			Gln636-Gly269	2.78
			Gln636-Gln295	2.65
			Gln636-Ala296	2.68
			Arg654-Ser208	2.55
6	hRBP 4	234.62	Asp31-Pro992	2.66
			Glu49-His293	2.83
			Glu49-Thr294	2.72
			Gln52-Gln213	2.30
			Asn66-His862	2.29
			Asn66-Arg864	2.21
			Thr76-Gln213	2.91
			Lys89-Leu214	2.29
			Lys89-Val216	2.53
			Trp91-Leu214	2.06
			Lys99-Ser231	2.73
			Gln149-Thr953	1.06
			Arg153-Thr953	1.79
			Arg153-Ser956	1.68
			Arg163-Phe998	2.70
			Arg163-Thr1001	2.85
			Arg166-Pro992	2.96
			Arg166-Thr997	2.19
			Leu167-Ser951	1.98
			Leu167-Gly958	2.68
			Leu167-Thr997	2.62
			Leu167-Thr999	2.63
7	hIGFBP 7	323.81	Glu67-Arg802	2.83
			Gln70-Arg802	2.70
			Arg74-Ser756	2.94
			Leu153-His805	2.76
			Pro154-His804	2.85
			Pro154-His805	2.42
			Arg189-Leu745	2.12
			Glu213-Gln746	1.94
			Glu213-Glu750	2.51
			Asn265-Gln896	1.29
			Asn265-Gly897	2.93
			Phe266-Asn870	2.56
			Phe266-His895	2.78
			Phe266-His900	2.51
			His272-Asp747	1.49
			His272-Leu829	1.93
			Gln273-Leu829	2.89
			Ser302-Arg827	2.63
			Asn304-Pro740	2.29
			Asn304-Pro824	1.65
			Asn304-Pro825	2.01
8	hCystatin C	295.47	Tyr62-Pro983	2.91
			Val68-Ser1006	1.69
			Arg70-Gln1040	2.83
			Arg70-Glu1046	2.30
			Thr74-Glu1046	3.00
			Gln77-Gln1048	2.12
			Pro78-Val949	2.57
			Asp81-Pro1032	2.04
			Phe85-Arg1008	2.82
			Asp87-Leu1049	3.00
			Phe99-Pro1005	2.34
			Ile101-Gln1004	1.94
			Pro105-His986	2.72
			Gly108-Tyr987	2.55
			Gly108-Val988	2.47
			Met110-Tyr977	2.04
			Leu112-His955	2.66
			Leu112-Gln1004	1.22
			Ser115-Leu1038	2.38
9	hPodocin	305.82	Leu105-Gly153	2.47
			Phe112-Arg207	2.11
			Lys126-His330	2.45
			Gln129-Asp251	2.21
			Tyr131-Thr333	2.44
			Tyr162-Glu329	2.48
			His163-Thr327	2.76
			His163-Glu329	2.77
			Lys164-Glu329	2.97
			Lys164-Gly331	2.85
			Asp166-Gly312	2.61
			Asp166-Gln314	2.98
			Asp166-Thr333	1.95
			Arg168-Thr333	2.91
			Arg168-Gln335	2.60
			Gln170-Asn281	2.69
			Thr171-Glu309	2.36
			Tyr194-Leu392	2.57
			His209-Gln283	2.17
			Ser211-Gln283	2.94
			Lys212-Gln283	2.83
			Ser336-Gln532	2.50
10	hBeta arrestin 1	254.64	Lys17-Ile742	2.60
			Lys17-Arg828	2.93
			Lys49-Asp747	2.50
			Tyr63-Gln277	2.68
			Leu73-Gln1148	2.50
			Thr74-Thr275	1.48
			Thr74-Trp289	2.94
			Phe75-His320	2.68
			Lys138-Thr287	2.51
			Lys160-Phe1145	2.51
			Lys160-Pro1147	2.08
			Asn245-Gln277	2.96
			Asn245-Leu279	2.57
			Asn245-Glu318	2.95
			Tyr249-Gln283	2.94
			Asp390-Thr710	2.33
			Ala392-Asn708	2.48
			Gln394-Pro694	2.54
			Arg395-Trp707	2.88
11	hVANGL2	329.28	Leu128-Thr233	2.02
			Leu132-Met147	2.99
			Leu133-Arg1143	2.10
			Arg135-Met147	1.77
			Glu146-Leu137	2.40
			Ile150-Lys229	2.05
			Phe154-Pro227	1.49
			Leu181-Thr1001	2.52
			Arg203-Glu780	2.38
			Val235-Val991	1.68
			Leu236-Thr999	2.26
			Arg240-Tyr977	2.79
			Arg240-Leu1000	2.81
			Arg240-Thr1001	1.76
			Gln245-His986	2.38
			His265-Tyr977	2.48
			Pro467-Leu1038	1.61
			Asn470-His955	2.41
			Asn470-Gln1004	2.94
			Asn470-Pro1005	2.06
			Gly471-Gln1004	1.25
			Lys473-Gln1004	2.21
12	hDynamin 1	262.85	Ala27-Lys430	2.63
			Asp106-Gly258	2.19
			Arg107-Arg256	1.07
			Arg107-Ala257	2.11
			Thr111-Thr305	2.58
			Asn112-Glu309	2.30
			Asp147-Glu309	2.47
			Asp151-Lys364	2.99
			Asp158-Lys431	0.89
			Gln196-Glu503	2.23
			Arg217-Gly1215	2.65
			Arg217-Asp1218	2.21
			Glu221-Arg460	2.60
			Glu221-Asp1218	2.78
			Lys223-Asp1218	2.89
			Leu224-Pro491	2.42
			Arg228-Glu503	2.88
			Pro263-Gln1219	2.98
			Ser264-Gln1219	2.94
			His267-Gln1219	2.85
			His267-Asp1223	2.05
			Asp291-Arg1229	2.71
			Gln303-Trp378	2.94
13	hTENC1	269.73	Arg1271-Val151	2.89
			Arg1271-Ser322	2.82
			Leu1289-Leu778	2.84
			Leu1289-Leu829	2.69
			Leu1289-Leu1142	2.97
			Gln1293-Gln815	1.12
			Gln1293-Arg827	2.02
			Arg1297-Asp747	2.60
			Arg1297-Leu829	2.60
			Ser1304-Asp747	2.56
			Gln1321-Thr149	1.45
			Arg1335-Glu143	2.28
			Arg1336-Glu143	2.50
			Pro1339-Met147	1.65
			Pro1339-Thr149	2.84
			Thr1344-Phe1145	2.98
			Phe1345-Phe1145	2.91
			Gln1351-Arg828	2.68
			Arg1353-Arg828	2.57
			Pro1373-Gly145	2.17
			Thr1401-Pro1147	1.61

In the present study, there is no correlation between the PC properties and molecular docking analysis, since PC is an independent property. However, in the case of hNeph, the first four highest docking score proteins exhibited an instability index value greater than 40. Moreover, all the HDOCK docking scores of hNeph have exhibited more than -200 (kcal/mol) which might be due to larger molecular weight protein compared to that of mNeph.

## Discussion

Nishibori and coworkers have reported that nephrin is confined to SD which is mainly based on its cytosolic interaction with podocin protein [25]. Li and colleagues have reported that nephrin interacts with many other podocyte proteins as well as with SD protein and thus is involved in activating cell signaling pathways in podocytes [26]. In vivo (mouse) knockout experimental models have reported that the impairment of nephrin (or) podocin (or) neph1 expression is enough to interrupt SD formation and stimulates severe disease within days and also even in utero [27]. Nephrin interacts with beta arrestin, which in turn stimulates endocytosis in both clathrin- and dynamin-dependent modes of action [27]. Coward and coworkers have demonstrated that nephrin is essential for the insulin action on glomerular podocytes of humans [28]. Similarly, Soda and colleagues have reported that dynamin has been complexed with nephrin in an indirect way during its own endocytosis processes [29]. Rocque and colleagues reported that the deficiency of VANGL2 activity which plays a vital role in the planar cell polarity (PCP) pathway leads to the elevated surface expression of nephrin in podocytes in mice (in vivo) experimental model [19]. C1-Ten has been known as nephrin tyrosine phosphatase, which is reported to be up-regulated in DN and also involved in the stimulation of podocyte hypertrophy [21]. Shree and coworkers have demonstrated the interaction of MMP 9 with nephrin using the protein-protein docking method [30]. In the present study, seven selected proteins (hMMP 2, hMMP 9, hRBP 3, hRBP 4, hKLK, hUromodulin, and hBeta arrestin 1) have shown an instability index value lesser than 40 that demonstrate these said proteins are unstable in nature [24]. Similarly, in the current investigation, all the chosen proteins (except hNeph) have exhibited the lowest grand average of hydropathicity of the protein (GRAVY) value which indicates these said proteins are known to have good interaction with water molecules [24].

With regard to the HDOCK (protein-protein) docking analysis, in the current investigation, all the docking scores have exhibited more than -200 kcal/mol with hNeph. On the other hand, all the docking scores have exhibited less than -200 kcal/mol (except for hMMP 9 and hKLK 1) with mNeph. In the present investigation, hMMP 9, with the following amino acid residues, namely, Tyr149, Gly186, Leu188, Ala189, His190, Ala191, Phe192, Pro193, Tyr218, His226, Gln227, His230, Asp235, His236, Pro246, Met247, and Tyr248, interacts with hNeph. These results were on par with earlier reports where Pro246 and Met247 amino acid residues of MMP 9 interact with nephrin using the ClusPro (protein-protein) docking method [30]. To the best of our knowledge, no other proteins have been reported to interact with hNeph (or) mNeph so far. 

Limitations and future recommendations

The current study findings are based on the HDOCK (protein-protein) docking analysis which provides new insight about these 13 proteins, namely, hMMP 2, hMMP 9, hRBP 3, hRBP 4, hKLK 1, hUromodulin, hIGFBP 7, hCystatin C, hPodocin, hBeta arrestin 1, hVANGL2, hDynamin 1, and hTENC1, as modular proteins of kidney function, and moreover, it is considered as a preliminary research work. Furthermore, in vitro (cell-based) and in vivo (mouse or rat animal model) experiments are needed to confirm these 13 ligands (proteins) as good modulating action against both hNeph and mNeph.

## Conclusions

The present HDOCK (protein-protein) investigation has shown that all 13 proteins dock very effectively with the two target proteins, namely, hNeph and mNeph. Interestingly, in the case of hNeph, all the HDOCK docking scores have exhibited more than -200 (kcal/mol), which might be a larger molecular weight protein compared to that of mNeph. The protein-protein (HDOCK) docking analysis has shown that hVANGL2 exhibited the maximum docking score of -329.28 (kcal/mol) with the target protein hNeph. Thus, the current finding highlights the interactions of hNeph and mNeph with 13 chosen proteins, which may help in renal disease management.

## References

[1] Kostovska I, Trajkovska KT, Cekovska S, Spasovski G, Labudovic D (2016). Nephrin and podocalyxin - new podocyte proteins for early detection of secondary nephropathies. Bantao.

[2] Brinkkoetter PT, Ising C, Benzing T (2013). The role of the podocyte in albumin filtration. Nat Rev Nephrol.

[3] Welsh GI, Saleem MA (2010). Nephrin-signature molecule of the glomerular podocyte?. J Pathol.

[4] Kawachi H, Fukusumi Y (2020). New insight into podocyte slit diaphragm, a therapeutic target of proteinuria. Clin Exp Nephrol.

[5] Tryggvason K (1999). Unraveling the mechanisms of glomerular ultrafiltration: nephrin, a key component of the slit diaphragm. J Am Soc Nephrol.

[6] Fayed A, Rahman Tohamy IA, Kahla H, Elsayed NM, El Ansary M, Saadi G (2019). Urinary podocyte-associated mRNA profile in Egyptian patients with diabetic nephropathy. Diabetes Metab Syndr.

[7] El-Sebai A, Yousef M, Shaaban MA, El-Farsy MS (2021). Urinary nephrin as a marker of glomerular diseases in pediatric patients. GEGET.

[8] Kondapi K, Kumar NL, Moorthy S, Silambanan S (2021). A study of association of urinary nephrin with albuminuria in patients with diabetic nephropathy. Indian J Nephrol.

[9] Kandasamy Y, Smith R, Lumbers ER, Rudd D (2014). Nephrin - a biomarker of early glomerular injury. Biomark Res.

[10] Li SY, Huang PH, Yang AH (2014). Matrix metalloproteinase-9 deficiency attenuates diabetic nephropathy by modulation of podocyte functions and dedifferentiation. Kidney Int.

[11] Hirata T, Fan F, Fan L (2023). Knockout of matrix metalloproteinase 2 opposes hypertension- and diabetes-induced nephropathy. J Cardiovasc Pharmacol.

[12] Tsai YL, Liu CW, Huang SF (2020). Urinary fatty acid and retinol binding protein-4 predict CKD progression in severe NAFLD patients with hypertension: 4-year study with clinical and experimental approaches. Medicine (Baltimore).

[13] Härma MA, Dahlström EH, Sandholm N, Forsblom C, Groop PH, Lehto M (2020). Decreased plasma kallikrein activity is associated with reduced kidney function in individuals with type 1 diabetes. Diabetologia.

[14] Thielemans R, Speeckaert R, Delrue C, De Bruyne S, Oyaert M, Speeckaert MM (2023). Unveiling the hidden power of uromodulin: a promising potential biomarker for kidney diseases. Diagnostics (Basel).

[15] Wang S, Chi K, Wu D, Hong Q (2021). Insulin-like growth factor binding proteins in kidney disease. Front Pharmacol.

[16] Benoit SW, Ciccia EA, Devarajan P (2020). Cystatin C as a biomarker of chronic kidney disease: latest developments. Expert Rev Mol Diagn.

[17] ElShaarawy A, Abdelmoneim BM, Abdelhameed BS, Abdelsattar HA, Shadad E (2019). Urinary podocin level as a predictor of diabetic kidney disease. J Nephropathol.

[18] Xu H, Li Q, Liu J (2018). β-Arrestin-1 deficiency ameliorates renal interstitial fibrosis by blocking Wnt1/β-catenin signaling in mice. J Mol Med (Berl).

[19] Rocque BL, Babayeva S, Li J (2015). Deficiency of the planar cell polarity protein Vangl2 in podocytes affects glomerular morphogenesis and increases susceptibility to injury. J Am Soc Nephrol.

[20] Khalil R, Koop K, Kreutz R, Spaink HP, Hogendoorn PC, Bruijn JA, Baelde HJ (2019). Increased dynamin expression precedes proteinuria in glomerular disease. J Pathol.

[21] Lee J, Koh A, Jeong H, Kim E, Ha TS, Saleem MA, Ryu SH (2017). C1-Ten is a PTPase of nephrin, regulating podocyte hypertrophy through mTORC1 activation. Sci Rep.

[22] Radhakrishnan N, Prabhakaran VS, Wadaan MA, Baabbad A, Vinayagam R, Kang SG (2023). STITCH, physicochemical, ADMET, and in silico analysis of selected Mikania constituents as anti-inflammatory agents. Processes.

[23] Maaz M, Dilshad E, Suliman Suliman, Ashraf NM (2022). Identification of anti-inflammatory metabolites from Trigonella foenum-greacum using computational approaches. Curr Trend Omics.

[24] Vishnupriya N, Narayanaswamy R (2024). Human intelectin-1 (hITL-1) as modulator of metabolic syndrome (MetS): an in silico study. J Pharm Bioallied Sci.

[25] Nishibori Y, Liu L, Hosoyamada M (2004). Disease-causing missense mutations in NPHS2 gene alter normal nephrin trafficking to the plasma membrane. Kidney Int.

[26] Li X, Chuang PY, D'Agati VD (2015). Nephrin preserves podocyte viability and glomerular structure and function in adult kidneys. J Am Soc Nephrol.

[27] Martin CE, Jones N (2018). Nephrin signaling in the podocyte: an updated view of signal regulation at the slit diaphragm and beyond. Front Endocrinol (Lausanne).

[28] Coward RJ, Welsh GI, Koziell A (2007). Nephrin is critical for the action of insulin on human glomerular podocytes. Diabetes.

[29] Soda K, Balkin DM, Ferguson SM (2012). Role of dynamin, synaptojanin, and endophilin in podocyte foot processes. J Clin Invest.

[30] Shree P, Srivastava S, Chaube R, Tripathi YB (2022). Protein-protein docking interaction of nephrin protein with the expression of MMP-9: an anti-inflammatory agent. Res J Biotechnol.

